# Upcycling Agri-Food Side-Streams via *Tenebrio molitor* Rearing: Growth Performance, Nutritional Composition, and Frass Quality of Larvae

**DOI:** 10.3390/foods15091478

**Published:** 2026-04-23

**Authors:** Labinot Kryeziu, Fabiola Neitzel, Rolf-Alexander Düring, Rainer Waldhardt, Martin Rühl

**Affiliations:** 1 Division of Landscape Ecology and Landscape Planning, Institute of Landscape Ecology and Resource Management (ILR), Research Centre for Biosystems, Land Use and Nutrition (iFZ), Justus Liebig University Giessen, 35392 Giessen, Germany; rainer.waldhardt@umwelt.uni-giessen.de; 2 Branch for Bioresources, Fraunhofer Institute for Molecular Biology and Applied Ecology (IME), 35392 Giessen, Germany; fabiola.neitzel@ime.fraunhofer.de; 3 Institute of Soil Science and Soil Conservation, Research Centre for Biosystems, Land Use and Nutrition (iFZ), Justus Liebig University Giessen, 35392 Giessen, Germany; rolf-alexander.duering@umwelt.uni-giessen.de; 4 Institute for Food Chemistry and Food Biotechnology, Justus Liebig University Giessen, 35392 Giessen, Germany

**Keywords:** agri-food, side-streams, valorization, mealworm, *Tenebrio molitor*, Kosovo

## Abstract

Insect bioconversion can transform agri-food side-streams into insect biomass while returning nutrients in the form of frass. This study evaluated *Tenebrio molitor* reared on wheat bran and on substrates in which bran was partially replaced with agri-food side-streams produced in Kosovo, including brewer’s spent grain, brewer’s spent yeast, apple and grape pomace, and surplus vegetables. Growth performance, larval composition, and frass nutrient composition were assessed across seven treatments. Larvae reared on wheat bran combined with brewer’s spent grain and melon as wet feed achieved the highest larval weight. Substrates containing wheat bran, apple pomace, and brewer spent yeast also supported high larval weights, but with a higher feed conversion ratio. In contrast, larvae reared on wheat bran, grape pomace, and brewer’s spent yeast showed the lowest larval weights. The use of potatoes as wet feed was associated with a longer development period. Larval proximate composition remained similar across treatments, with crude protein contents of 52–56% DM and only limited variation in fat and ash content. Overall, several tested side-streams supported larval growth comparable to wheat bran while generating nutrient-containing frass.

## 1. Introduction

Global demand for food and feed is expected to increase substantially over the coming decades, placing growing pressure on agri-food systems operating under land and water constraints [1]. Current agricultural production systems, particularly those dependent on intensive livestock farming, require substantial land and water inputs and contribute significantly to greenhouse gas emissions and biodiversity loss, raising concerns about their long-term environmental sustainability under climate change and limited opportunities for further agricultural land expansion [1,2]. At the same time, large quantities of organic residues are generated along agri-food value chains but remain insufficiently valorized, leading to nutrient losses and additional environmental burdens [1,3]. Together, these challenges underscore the need for alternative protein production pathways that reduce resource use while enabling the valorization of agri-food side-streams [1].

In this context, insects offer a well-established capacity to convert organic residues into biomass within circular food-system frameworks [2,4]. The yellow mealworm (*Tenebrio molitor* L.; hereafter, mealworm) is a widely studied insect for food and feed applications, and studies have shown that it can utilize a broad range of agri-food side-streams and organic residues, producing larval biomass and frass that enable the valorization of side-streams otherwise contributing to waste [5,6,7,8,9]. Recent studies have further examined the effects of alternative substrates and agro-industrial side-streams on mealworm rearing. Jankauskienė et al. [10] showed that mealworms reared on substrates including brewers’ spent grain and other side-streams maintained high nutritional value, although protein content did not differ significantly among rearing conditions. Mahmoud et al. [11] similarly reported that diet composition significantly affected larval growth, development, and macronutrient composition. More recently, Ferri et al. [12] found that supplementing wheat bran with carob or brewer’s spent grain improved larval growth performance and feed conversion efficiency, while the nutritional composition of the resulting insect meals remained largely comparable across treatments. Together, these studies confirm that substrate formulation is an important factor in mealworm production, although the effects of specific side-streams on performance and composition are context dependent. Accordingly, substrate formulation remains a key factor influencing mealworm performance and the composition of the resulting larval biomass [7,13,14]. Dried mealworm larvae were authorized as a novel food in the European Union in 2021, followed by authorization of a UV-treated, vitamin-D-enriched mealworm powder in 2025 for use in staple foods [15,16].

Mealworm-based valorization of side-streams is particularly relevant in contexts such as Kosovo, where substantial volumes of organic residues remain underutilized within existing waste pathways. Kosovo’s agri-food sector is economically important but constrained by highly fragmented agricultural holdings, with 92.7% of farms cultivating less than 5 ha and together managing approximately 60% of the country’s arable land [17]. This structural context is reflected in a persistent imbalance between agricultural imports and exports, with an import–export ratio of approximately 10:1 [18]. At the same time, Kosovo is associated with a range of underutilized agri-food side-streams across its main value chains, including potential wheat-milling side-streams (e.g., bran), brewery residues (spent grain and spent yeast) [19], side-streams typically associated with apple production, the country’s dominant fruit crop [18], and grape pomace from the concentrated viticulture sector in Rahovec, which produces around 9–12 million liters of wine annually [20]. In addition, vegetable production, particularly peppers and potatoes, which represent key vegetable crops, is associated with seasonal surpluses and post-harvest losses, while cucurbits such as melon may contribute further residues during the summer harvest period [18].

The present study examines the growth performance, larval composition, and frass characteristics of mealworm larvae reared on wheat bran and on substrates in which bran is partially replaced with regionally abundant agri-food side-streams, evaluating whether such formulations can sustain larval performance and composition relative to the control.

## 2. Materials and Methods

The study was conducted from autumn 2022 to late summer 2023 at the Fraunhofer IME facilities in Giessen, Germany.

### 2.1. Preparation of Feeding Substrates

Side-streams from agri-food activities in Kosovo were used as substrate ingredients: brewery spent grain (BSG), brewery spent yeast (BSY), apple pomace (AP), grape pomace (GP), and wheat bran (WB), which served as the sole substrate in the control treatment (S1) and as the primary basal component in all other treatments (S2–S7). The moist side streams (BSG, BSY, AP, and GP) were stored frozen prior to processing. Wheat bran was used as received without further processing. The other substrate ingredients intended for dry-feed formulation were oven-dried at 50 °C to constant weight and ground using a Thermomix (Vorwerk, Wuppertal, Germany) to obtain a fine, homogeneous particle structure; no sieving step was applied. Fresh vegetable side streams (pepper, melon, and potato) were chopped and used as wet feed. Pepper and potato residues were selected as widely cultivated vegetable crops within Kosovo’s agricultural sector [21]. Pepper was used as the primary wet feed across treatments, while potato represents a commonly used moisture source in mealworm rearing systems [22,23]. Melon was included as a representative perishable crop with limited post-harvest storage stability [24].

Substrate treatments (S1–S7) were prepared by combining the respective ingredients in predetermined ratios selected for experimental comparison (Table 1). Treatments S2–S4 differed only in wet-feed type, whereas treatments S5–S7 included brewery spent yeast combined with fruit pomace. Substrate treatments (S1–S7) served as the primary dry feed, while wet feed was supplied to provide moisture. Larvae reared on these treatments are referred to as L1–L7, with corresponding frass labeled F1–F7.

### 2.2. Rearing for Growth Performance

Mealworm eggs were sourced from Nertus GmbH (Walzbachtal, Germany) and reared in a climate chamber (ICH750eco, Memmert, Schwabach, Germany) at 27 °C, 70% relative humidity, under constant darkness.

For growth performance assessment, three replicate boxes (10 × 10 × 10 cm) per treatment were set up with 350 eggs and 10 g substrate, with each box serving as an independent biological replicate (n = 3); the small number of replicates per treatment (n = 3) may limit the statistical power of the comparisons and should be considered when interpreting the results. Additional dry substrate was supplied in four increments during rearing (10 g in Week I, 30 g in Week III, 40 g in Week VI, and 20 g in Week VIII), resulting in 100 g total dry feed per box. Wet feed (100 g total per replicate) was supplied three times per week (Monday, Wednesday, Friday) from Week IV onward. Feeding started with portions of approximately 1.5 g per feeding event and was progressively increased in line with larval development to approximately 10 g per feeding in later stages. Feeding was continued until the onset of pupation. Uneaten wet-feed residues were removed prior to each feeding and replaced with fresh material to prevent spoilage. Beginning in Week IV, 50 larvae per box were randomly sampled and weighed weekly. The trial ended at first pupation. At harvest, 50 larvae per box were weighed after a 24 h starvation period, and 50 pupae per box were collected and weighed immediately after pupation.

Substrate reduction (SR), waste reduction index (WRI), feed conversion ratio (FCR), and efficiency of conversion of ingested feed (ECI) were calculated according to Equations (1)–(4). FCR was calculated on an as-fed basis using larval biomass expressed as fresh matter, whereas SR, WRI, and ECI were calculated on a dry-matter basis. Because these metrics are calculated on different bases, they are not directly comparable with each other. Wet feed was excluded from all calculations due to its negligible dry-matter contribution relative to the substrate.

(1) $$SR\left(\%\right)=\frac{S-F}{S}\times 100$$

(2) $$WRI\;\left(\%\;day^{-1}\right)=\frac{SR}{t}$$

(3) $$FCR=\frac{S}{\Delta B}$$

(4) $$ECI\left(\%\right)=\frac{\Delta B}{S-F}\times 100$$

In the equations, S denotes the total substrate supplied (g), F the frass produced (g), ΔB the larval biomass gain (g), and t the rearing duration (days).

### 2.3. Rearing for Chemical Analyses

For compositional analyses, three replicate boxes (16.5 × 19.5 × 9.5 cm) per substrate treatment were stocked with 100 adult beetles. Adults from the routinely maintained Fraunhofer IME colony were allowed to oviposit for seven days before removal. Boxes received 450 g of dry substrate and 400 g of wet feed, supplied in gradually increasing portions under the same feeding frequency and environmental conditions as described for the growth trial in Section 2.2. Larvae from each replicate box were frozen, ground under liquid nitrogen, and freeze-dried. Powders were stored at −20 °C until analysis. Adult beetles were used exclusively for oviposition to obtain eggs, whereas all growth performance measurements and chemical analyses were conducted on larvae.

### 2.4. Chemical Analyses

Unless otherwise stated, chemical analyses were performed in triplicate. Amino acids and C:N were analyzed in duplicate, and mineral composition was determined once per pooled sample. For chemical analyses, larval and side-stream samples were freeze-dried (Delta LSCplus, Martin Christ Gefriertrocknungsanlagen, Osterode am Harz, Germany) and ground to a fine powder. The composition of substrate treatments (S1–S7) was calculated from the analytically determined ingredient values using the respective mixing ratios. Proximate and elemental values are expressed as % dry matter (DM), moisture as % fresh weight, and mineral concentrations as mg/kg DM.

#### 2.4.1. Proximate Composition

Moisture, ash, crude fat, crude protein, and crude fiber were determined as described previously [25,26]. Moisture was measured using a MA35 moisture analyzer (Sartorius, Göttingen, Germany). Ash was determined by pre-ashing over a Bunsen burner and then combusting at 550 °C for 6 h in a muffle furnace (L9/11, Nabertherm, Lilienthal, Germany). Crude protein content was analyzed by the Kjeldahl method [27,28] using a Behrotest S5 digestion unit (behr Labor-Technik, Düsseldorf, Germany) and a TitroLine 5000 titration system (SI Analytics, Mainz, Germany), applying a nitrogen-to-protein conversion factor of 6.25 for plant substrates and 5.9 for mealworm larvae, based on their amino acid composition. Crude fat was extracted after acid hydrolysis (Weibull–Stoldt method) with petroleum ether using a Soxtherm-type system (Behr E6, behr Labor-Technik, Düsseldorf, Germany). Crude fiber was analyzed by the Scharrer–Kürschner method, involving oxidative acid digestion and ashing at 700 °C [29].

#### 2.4.2. Fatty Acid Analysis

Fatty acids were analyzed from lipid extracts obtained during crude-fat determination. Lipids were dissolved in iso-octane and converted to fatty acid methyl esters (FAMEs) by a two-step transesterification procedure using methanolic NaOH followed by boron trifluoride in methanol (BF_3_–MeOH). FAMEs were analyzed by GC–MS (7890B GC coupled to 5977B MSD, Agilent Technologies, Waldbronn, Germany) using a VF-WAX ms column (30 m × 0.25 mm, 0.25 µm film thickness) under conditions described previously [26,30]. Fatty acids were identified using a 37-component FAME mix (Supelco, Bellefonte, PA, USA) and expressed as relative percentages of total identified FAMEs.

#### 2.4.3. Amino Acid Analysis

Amino acids were analyzed as previously described [25,26,31]. Freeze-dried samples (20–40 mg) were hydrolyzed under three conditions: (i) in 6 M HCl with 1 g L^−1^ phenol, (ii) after performic acid oxidation for cysteine and methionine, and (iii) in 5 M NaOH with 0.1% phenol for tryptophan. Hydrolysates were adjusted to pH 2.2, diluted with citrate buffer, and filtered (0.45 µm) before analysis. Analyses were carried out in duplicate for each treatment and quantified on an amino acid analyzer (S433, Sykam Chromatographie Vertriebs GmbH, Fürstenfeldbruck, Germany) with post-column ninhydrin derivatization (570 nm; 440 nm for proline) using Chromstar software (version 7; Sykam Chromatographie Vertriebs GmbH, Fürstenfeldbruck, Germany). Amino acid profiles are expressed as relative percentages of total identified amino acids.

#### 2.4.4. Elemental and Mineral Composition

Total carbon (C; DIN EN 15936:2012-11) [32] and total nitrogen (N; DIN EN 16168:2012-11) [33] were analyzed using an Elemental Unicube analyzer (Elementar Analysensysteme GmbH, Langenselbold, Germany). Macro- and trace elements (taking into account both essential and non-essential elements) (As, Ca, Cd, Cr, Cu, Fe, K, Mg, Mn, Ni, P, Pb, S, Zn) were quantified by inductively coupled plasma–optical emission spectrometry (ICP–OES; 720-ES, Varian, Darmstadt, Germany) after microwave-assisted EDTA extraction (DIN ISO 11466/DIN EN 16174) [34,35].

### 2.5. Statistical Analysis

Data were analyzed in R (version 4.5.3). Performance and compositional parameters determined from three biological replicates were analyzed using one-way analysis of variance (ANOVA), followed by Tukey’s honestly significant difference (HSD) test at α = 0.05. Assumptions of normality and homogeneity of variance were evaluated using the Shapiro–Wilk and Levene’s tests, respectively, for variables with three biological replicates. Variables determined in duplicate (e.g., C total, N total, C:N ratio, and amino acid composition) were summarized descriptively as mean values without inferential statistical testing. Substrate composition data, obtained from technical replicates, are presented descriptively (mean ± SD) without statistical comparison. Weekly growth data (mean larval weight per substrate treatment) and mineral data were also summarized descriptively.

## 3. Results and Discussion

### 3.1. Composition of Substrate Treatments

The control substrate treatment (S1) showed a composition consistent with reported values for wheat bran [36,37,38] (Table 2). Compared with S1, brewery-residue-containing treatments had higher crude protein contents, whereas fat, fiber, ash, and elemental composition varied among substrate treatments according to formulation. Among wet feeds, pepper and melon showed higher moisture contents than potato (Table 2).

Beyond proximate composition, fatty acid profiles varied among substrate treatments (Table S1). In comparison to all other substrates, S1 showed a higher proportion of linoleic acid and total polyunsaturated fatty acids (PUFA), which is consistent with published wheat bran data [39,40]. Treatments S2–S5 tended to show higher proportions of saturated (SFA) and monounsaturated fatty acids (MUFA) and lower PUFA levels than S1. In contrast, S6 showed higher PUFA levels than S2–S5, whereas S7 exhibited the highest n-6/n-3 ratio among the substrate treatments (Table S1).

Amino acid profiles differed among treatments (Table S2). Total essential amino acids (EAA; defined for humans) ranged from 35.1% (S1) to 38.3% (S6). Accordingly, the EAA/NEAA ratio increased from 0.54 (S1) to 0.62 (S6) (Table S2).

Macro and trace element composition varied among substrate treatments and wet feeds (Table 3). Potassium (K) and phosphorus (P) were the predominant macro elements across substrates, whereas trace elements occurred at substantially lower concentrations. Some elements nonetheless varied among treatments; for example, calcium ranged from 905 mg/kg DM (S6) to 2278 mg/kg DM (S7), and iron was highest in S7 (345 mg/kg DM) compared with 116–160 mg/kg DM in the other substrates. Among wet feeds, melon and potato showed higher potassium and phosphorus concentrations than pepper, whereas calcium was highest in pepper, and iron was highest in potato. Most other elements occurred at comparable levels across the wet feeds.

### 3.2. Larval Growth Performance and Nutritional Composition

#### 3.2.1. Larval Growth Performance

**Figure 1:**
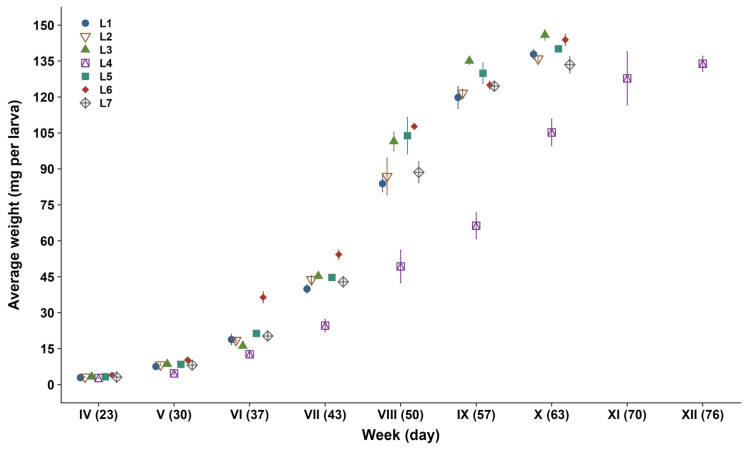
Weekly mean larval weight (mg per larva; mean ± SD) of mealworms (L1–L7) reared on seven different substrates (S1–S7).

By day 63, all treatments except L4 had reached pupation (Figure 1), with L4 larvae remaining in the larval stage until day 76. L4 larvae exhibited slower growth and stayed the lightest until pupation. These observations are consistent with known variation in dietary nutrient composition influencing insect growth and developmental timing [41]. Development times observed in this study align with previous reports for mealworms reared on wheat bran-based substrates supplemented with agri-food side-streams, where pupation has been observed between approximately 6 and 12 weeks, depending on substrate composition and moisture supply [42,43,44,45,46]. The delayed development observed in L4, reared on the same substrate as L2–L3 but with potato used as wet feed, is consistent with reports showing faster larval growth and shorter development times when high-moisture vegetables are used as water sources [14,47,48].

At pupation, final larval weight (FLW) differed among treatments. In holometabolous insects, growth is restricted to the pre-metamorphic larval stages, and final larval weight plays a key role in the developmental transition that can influence adult size and performance [49]. L3 and L6 are significantly heavier larvae than L7, while L1, L2, and L5 showed intermediate values; L4 did not differ from L7 (Table 4). These values of 133–146 mg are consistent with reports for mealworms reared on wheat bran-based substrates supplemented with agri-food side-streams, for which FLWs of 76–160 mg have been reported, with variation largely attributed to differences in substrate composition, moisture supply, strain, and rearing temperature [13,47,50,51,52,53]. Among the BSG-based treatments, S3 differed from S2 only in wet feed (melon vs pepper) and produced the highest FLW, whereas S2 remained comparable to the wheat bran control (S1). Although melon and pepper provided similarly high moisture, melon exhibited a substantially higher C:N ratio (51 vs. 22; Table 2); this difference may have contributed to the higher larval weight observed in S3. In contrast, L7 had the lowest FLW among the treatments. Higher FLWs have been reported in studies using diets enriched with protein-rich side-streams, such as high-protein formulations or brewer’s spent grain supplemented wheat bran diets, with FLWs ranging from 146.9 to 196.7 mg [45,54]. In addition to larval growth, substrate utilization differed among treatments, with L1 showing the highest substrate reduction (SR) and waste reduction index (WRI), while the lowest values were observed for L6 and L4, respectively (Table 4).

Pupal weight (PW) varied across treatments, from 118 mg (L6) to 135 mg (L3). Because pupal weight is closely associated with adult reproductive potential, it is a reliable indicator of larval dietary quality [55]. L6 and L7 had the lowest pupal weights, though they were not significantly different from the control (L1: 123 mg), which had intermediate values. These values are consistent with published data, where pupal weights typically range from 117 to 161 mg, depending on substrate composition and rearing conditions [13,43,45,46].

Feed conversion ratio (FCR) ranged from 2.6 (L3) to 3.3 (L6), with intermediate values in the other treatments (Table 4). The efficiency of conversion of ingested feed (ECI) varied from 37.2% (L1) to 43.4% (L3 and L5), with L6 showing a high ECI (42.7%) despite its higher FCR. These values are consistent with those commonly reported for mealworms, where FCR and ECI typically vary depending on substrate composition and nutrient balance [13,48,56]. L6, reared on S6, which had the highest fiber content among all treatments (15.57% DM, Table 2), showed the highest FCR and lowest SR, consistent with reports that higher fiber intake reduces food assimilation [57]. L4, which used potato as wet feed, had the highest FCR and the lowest WRI compared to L1 (control) and L2–L3 (reared on the same dry substrate as L4). This is in line with previous studies on potato-rich substrates, where limited starch digestibility and glycoalkaloids have been discussed as potential contributing factors [13].

#### 3.2.2. Larval Nutritional Composition

Larval crude protein content varied from 52.6 to 56.3% (L1–L4) (Table 5). Ash content was between 3.5 and 3.9% (L1–L4), with slightly higher values in L3 and L4. Fat content decreased in treatments with alternative side streams, from 28.6 to 34.2% (L4–L1), with the highest fat content observed in L1. Fiber content ranged from 5.3 to 6.8% (L1–L4), highest in L4, with L3 and L6 showing higher fiber than other treatments. Total carbon (C) and nitrogen (N) contents remained nearly unchanged across treatments. These values fall within the proximate composition ranges previously published [7]. This pattern of relatively stable protein content, with more noticeable variation in fat and fiber, aligns with studies indicating that larval protein content tends to remain stable across different substrates, whereas fat and fiber fractions vary more with substrate composition [13,42,58].

Larval fatty acid profiles were dominated by oleic (C18:1Δ9), linoleic (C18:2Δ9,12), and palmitic (C16:0) acids across all treatments (Table 6), consistent with previous reports [59,60,61,62]. Total SFA, MUFA and PUFA accounted for 22–24%, 38–49% and 27–38% of total fatty acids, respectively, values that fall within the ranges reported for mealworm larvae on cereal and side-stream-based substrates [7,63,64]. PUFA proportions were lowest in L6, which showed the highest MUFA content, while n-6/n-3 ratios were lowest in L2–L5 and highest in L7. As reported by Ruschioni et al. [61], the larval fatty acid profiles did not simply parallel the fatty acid composition of the substrates (Table S1). Substrate influenced the relative proportions of PUFA and MUFA, but the major fatty acid profile remained consistent across treatments.

**Figure 2:**
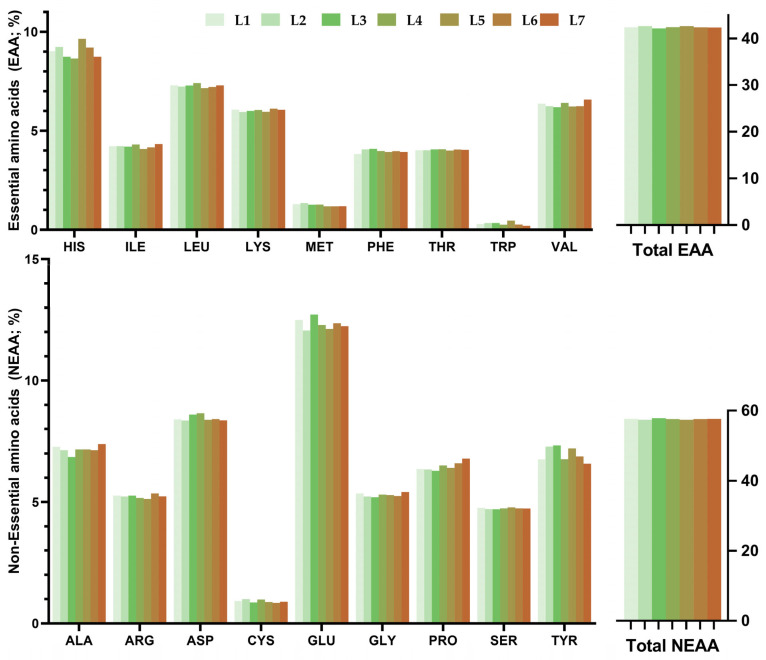
Amino acid profile (% of total identified amino acids) of larvae reared on different feed substrates. Mean values (n = 2) are presented.

Larval amino acid profiles were dominated by histidine (His), leucine (Leu), lysine (Lys) and valine (Val), which together accounted for the majority of essential amino acids (EAA) (Figure 2). Total EAA ranged from 42.1 to 42.6% across treatments. Among the non-essential amino acids (NEAA), glutamic acid (Glu), alanine (Ala), and aspartic acid (Asp) were the most abundant, with total NEAA content ranging from 57.4 to 57.9%. The EAA/NEAA ratio remained stable across treatments (0.73–0.74). The stability of the EAA/NEAA ratio across all treatments (0.73–0.74) suggests that mealworm larvae maintain compositional homeostasis in amino acid profiles regardless of substrate formulation within the range tested. These amino-acid profiles are consistent with previous reports, which show EAA profiles dominated by Leu, Lys, Val and His, and NEAA profiles dominated by Glu, Asp and Ala, with EAA/NEAA ratios typically around 0.7–0.75 [61,65].

In addition to amino acids and fatty acids, concentrations of macro and trace elements were broadly consistent across treatments (Table 7). Potassium (K) and phosphorus (P) were the predominant macro elements (7.3–8.7 g/kg DM and 7.7–9.4 g/kg DM, respectively), followed by sulfur (S, 3.2–3.6 g/kg DM) and magnesium (Mg, 2.0–2.4 g/kg DM), while calcium (Ca) occurred at lower levels (0.29–0.48 g/kg DM). Among trace elements, zinc (Zn), iron (Fe), copper (Cu), and manganese (Mn) ranged from 109 to 135, 60–66, 16–23, and 11–15 mg/kg DM, respectively, whereas nickel (Ni) and chromium (Cr) occurred only at low levels, and arsenic (As), cadmium (Cd), and lead (Pb) were below the limits of detection or quantification. This K–P dominance, with Fe and Zn as the main trace elements, agrees with mineral profiles of mealworm larvae reared on cereal and side-stream based substrates [7,22,54,66,67].

Among treatments sharing the same substrate (L2–L4), macro and trace element levels differed slightly, with L4 showing somewhat higher K, Mg and P, likely reflecting the greater dry-matter contribution from potato compared to pepper or melon. Despite marked differences in substrate mineral composition (Table 3), larval mineral content remained stable across treatments, consistent with reports of limited substrate-driven changes in macro and trace element content and low heavy-metal levels when safe side streams are used [22,54,67,68].

Overall, the macro and trace element profiles of larvae reared on Kosovo side stream substrates are consistent with those reported in the literature, with As, Cd, and Pb levels below detection or quantification limits. This agrees with studies showing low heavy metal concentrations in mealworms when larvae are reared on appropriately sourced side-stream feedstocks [7,69,70].

### 3.3. Composition of Mealworm Frass

Frass composition varied with treatments (Table 8). Moisture content ranged from 16 to 19%, while ash content varied from 5.1 to 7.6% (F4–F1). Total carbon (C) and nitrogen (N) contents were 35.6–38.6% and 2.0–3.2%, respectively, resulting in C:N ratios of approximately 12–18.

These values are consistent with published data for mealworm frass produced on cereal and side-stream based substrates, where C contents ranged from 39 to 50%, N contents from 2.5 to 5% and C:N ratios from 8 to 23 [71,72,73].

Macro elements in the frass (Table 9) were dominated by potassium (K) and phosphorus (P) at concentrations of 9.1–14.3 and 9.0–11.5 g/kg DM, respectively, followed by magnesium (Mg, 2.8–5.0 g/kg DM) and calcium (Ca, 1.0–3.0 g/kg DM), with sulfur (S) ranging from 2.1 to 2.6 g/kg DM. Among trace elements, iron (Fe), manganese (Mn), zinc (Zn), and copper (Cu) occurred at concentrations of tens to hundreds of mg/kg DM, whereas nickel (Ni) and chromium (Cr) were detected only at low levels. Arsenic (As) and cadmium (Cd) were below detection or quantification limits, and lead (Pb) remained below 1 mg/kg DM. This macro and micronutrient pattern is consistent with published characterization of mealworm frass as a nutrient-rich organic fertilizer containing appreciable N, P, K, Ca and Mg, as well as agronomically relevant levels of micronutrients when larvae are reared on safe side stream substrates [71,73,74,75].

Although frass composition did not directly mirror substrate composition, qualitative patterns across Table 2, Table 3, Table 8 and Table 9 suggest potential substrate-related effects. For example, substrates with higher total N were associated with higher frass N contents, while similar trends were observed for macro elements. However, these observations are descriptive and were not statistically evaluated. Previous studies report that, despite modification through insect metabolism, substrate composition can influence frass nutrient profiles [71,73,74,75,76].

Previous studies have reported that mealworm frass and other insect frasses can increase biomass production and/or nutrient uptake in crops under pot or field conditions, including lettuce, sunflower, spinach, barley and forage grasses [74,76,77,78,79,80,81]. Field trials conducted in Kosovo similarly reported increased chamomile biomass and plant height following application of mealworm frass produced from local side streams [82]. However, crop responses have been shown to depend on frass processing, application rate, and maturity or salinity constraints [71,74,76,79]. Taken together, these results indicate that Kosovo’s relevant agri-food side-streams can be upcycled through mealworm rearing into larval biomass for food and feed applications, while generating a nutrient-containing frass that warrants further agronomic evaluation within circular agri-food systems. However, economic and logistical aspects of side-stream collection, processing, and integration into rearing systems were not assessed in this study and require further investigation. In addition, frass characterization in this study focused on parameters relevant to its evaluation as a nutrient source, particularly total C, N, and macro- and trace element composition. Proximate composition (e.g., crude protein, fat, and fiber) was not determined, as it was beyond the analytical scope of this work; however, inclusion of these fractions would provide additional insight into the organic matter composition of frass and should be considered in future studies.

## 4. Conclusions

This study evaluated mealworm larvae reared on wheat bran and substrates partially replaced with regionally abundant agri-food side-streams from Kosovo. These included brewer’s spent grain and yeast, apple and grape pomace, and surplus or slightly damaged vegetables (pepper, melon, and potato) used as wet feed. Among treatments, L3, reared on S3 (wheat bran and brewer’s spent grain with melon), produced the heaviest larval weight. L6, reared on wheat bran, apple pomace, and brewer’s yeast with pepper, also achieved high weights but with a higher feed conversion ratio. L7 showed the lowest mean larval weight, with L4 showing a similar value, although these remained within ranges reported for mealworms reared on side-stream substrates. L4, reared on the same dry substrate as L2–L3 but with potato as wet feed, required a longer development period. Larval crude protein, fatty-acid profiles, amino-acid profiles, and elemental composition showed minimal variation across treatments. These results indicate that inclusion of side-streams did not substantially alter larval nutritional quality, while frass composition remained within the ranges observed across treatments. Frass generated from these substrates showed nutrient profiles consistent with reported mealworm frass, supporting its further evaluation as a potential organic fertilizer. These findings suggest that regionally available agri-food side-streams can be utilized as feed components in mealworm rearing, supporting larval growth comparable to the control while maintaining nutritional composition and producing nutrient-containing frass. Future studies should evaluate economic feasibility and logistical considerations to support practical implementation.

## Figures and Tables

**Table 1:** Composition of substrate treatments used in the feeding trials.

Treatment	Ingredients (Ratio, %)	Wet Feed
S1	WB (100)	Pepper
S2	WB (70), BSG (30)	Pepper
S3	WB (70), BSG (30)	Melon
S4	WB (70), BSG (30)	Potato
S5	WB (70), BSG (25), BSY (5)	Pepper
S6	WB (55), AP (25), BSY (20)	Pepper
S7	WB (60), GP (25), BSY (15)	Pepper

**Table 2:** Composition of substrate treatments and wet feeds (mean ± SD).

	S1	S2–S4	S5	S6	S7	Pepper	Melon	Potato
Moisture (%)	12.2 ± 0.3	11.0 ± 0.3	11.2 ± 0.2	11.1 ± 0.4	11.2 ± 0.1	90.4 ± 3.0	91.9 ± 1.8	71.4 ± 1.1
Ash (% DM)	4.3 ± 0.1	4.5 ± 0.1	4.6 ± 0.1	4.2 ± 0.1	5.5 ± 0.0	8.3 ± 0.2	3.8 ± 0.1	5.7 ± 0.1
Crude Protein (% DM)	17.7 ± 0.3	19.4 ± 0.1	20.7 ± 0.1	20.7 ± 0.2	21.6 ± 0.2	n.d.	n.d.	n.d.
Fat (% DM)	3.9 ± 0.2	6.0 ± 0.1	5.5 ± 0.1	2.9 ± 0.1	4.9 ± 0.1	n.d.	n.d.	n.d.
Fiber (% DM)	8.0 ± 0.4	12.3 ± 1.0	11.3 ± 0.9	15.6 ± 0.9	10.9 ± 0.2	n.d.	n.d.	n.d.
C total (% DM)	42.1	44.0	43.7	42.6	43.8	40.3	38.3	39.28
N total (% DM)	2.6	3.0	3.1	3.0	3.1	1.8	0.8	1.61
C:N Ratio	16.3	14.8	14.0	14.2	14.0	22.0	50.8	24.41

Note: n.d. = not determined. For C total, N total, and C:N ratio, only the mean is reported due to the limited number of replicates (n = 2).

**Table 3:** Macro and trace element concentrations (mg/kg DM) of substrate treatments and wet feeds.

	S1	S2–S4	S5	S6	S7	Pepper	Melon	Potato	LOD	LOQ
As	<LOD	<LOD	<LOD	<LOD	<LOD	<LOD	<LOD	<LOD	1.2	3.7
Ca	925	1594	1467	905	2278	2728	1016	499	10.9	33.2
Cd	<LOD	<LOD	<LOD	<LOD	<LOD	<LOD	<LOD	<LOQ	0.07	0.2
Cr	0.53	0.63	0.60	0.44	1.28	0.21	0.42	0.59	0.01	0.04
Cu	10.2	10.8	10.4	7.9	18.5	3.3	6.5	4.4	0.14	0.4
Fe	147.5	159.5	154.4	115.8	345.4	32.0	42.6	144.9	0.07	0.2
K	9207	6596	7370	9370	11,141	10,365	19,639	17,147	0.19	0.6
Mg	3616	3225	3223	2529	2788	1489	1219	1297	18.5	56.1
Mn	133.2	109.3	107.0	77.6	91.0	6.1	11.0	11.9	2.0	6.1
Ni	1.1	0.9	0.8	1.4	2.1	2.6	3.2	2.1	0.19	0.6
P	8194	7447	7966	8038	7985	1360	3392	3345	1.1	3.2
Pb	<LOD	<LOD	<LOD	<LOD	<LOQ	<LOD	<LOD	<LOD	0.10	0.3
S	1714	1921	1973	1757	1948	1035	1987	1480	4.1	12.4
Zn	60.9	64.7	62.6	41.1	45.4	10.2	17.0	10.5	0.01	0.04

Note: LOD = limit of detection; LOQ = limit of quantification. Values below limits are reported as <LOD or <LOQ.

**Table 4:** Growth performance of mealworm larvae reared on different feed substrates (mean ± SD).

	L1	L2	L3	L4	L5	L6	L7
FLW (mg)	136 ± 1 ^bc^	136 ± 1 ^bc^	146 ± 2 ^a^	134 ± 3 ^bc^	140 ± 1 ^ab^	144 ± 2 ^a^	133 ± 3 ^c^
SR (%)	35.6 ± 1.3 ^a^	30.4 ± 2.5 ^ab^	30.5 ± 2.7 ^ab^	31.1 ± 1.5 ^ab^	33.7 ± 2.4 ^ab^	28.3 ± 4.3 ^b^	31.1 ± 1.1 ^ab^
WRI (% day^−1^)	0.6 ± 0.0 ^a^	0.5 ± 0.0 ^abc^	0.5 ± 0.0 ^abc^	0.4 ± 0.0 ^c^	0.5 ± 0.0 ^ab^	0.5 ± 0.1 ^bc^	0.5 ± 0.0 ^abc^
PW (mg)	123 ± 3 ^bc^	122 ± 3 ^bc^	135 ± 1 ^a^	130 ± 5 ^ab^	130 ± 2 ^ab^	118 ± 2 ^c^	120 ± 2 ^c^
FCR	2.8 ± 0.2 ^ab^	2.8 ± 0.2 ^ab^	2.6 ± 0.1 ^b^	3.0 ± 0.1 ^ab^	2.8 ± 0.2 ^ab^	3.3 ± 0.3 ^a^	2.7 ± 0.1 ^b^
ECI (%)	37.2 ± 1.1 ^b^	41.3 ± 0.6 ^ab^	43.4 ± 1.4 ^a^	42.4 ± 3.5 ^ab^	43.4 ± 0.1 ^a^	42.7 ± 3.8 ^ab^	40.4 ± 0.3 ^ab^

Note: FLW = final larval weight; SR = substrate reduction; WRI = waste reduction index; FCR = feed conversion ratio; PW = pupal weight; ECI = efficiency of conversion of ingested feed. Different letters indicate significant differences (*p* < 0.05).

**Table 5:** Nutritional composition of mealworm larvae reared on different feed substrates (mean ± SD).

	L1	L2	L3	L4	L5	L6	L7
Ash (% DM)	3.5 ± 0.3 ^b^	3.7 ± 0.1 ^ab^	3.9 ± 0.2 ^a^	3.9 ± 0.1 ^a^	3.6 ± 0.0 ^ab^	3.7 ± 0.1 ^ab^	3.8 ± 0.1 ^ab^
Crude Protein (% DM)	52.6 ± 0.6	53.6 ± 2.1	54.1 ± 1.8	56.3 ± 2.4	56.0 ± 0.2	55.1 ± 1.8	55.7 ± 1.4
Fat (% DM)	34.2 ± 0.7 ^a^	31.9 ± 0.5 ^ab^	31.8 ± 1.7 ^ab^	28.6 ± 1.4 ^b^	30.4 ± 1.8 ^b^	31.9 ± 0.8 ^ab^	28.9 ± 1.7 ^b^
Fiber (% DM)	5.3 ± 0.1 ^c^	5.6 ± 0.1 ^c^	6.1 ± 0.1 ^b^	6.8 ± 0.1 ^a^	5.5 ± 0.2 ^c^	6.2 ± 0.1 ^b^	5.7 ± 0.3 ^c^
C total (% DM)	56.4	56.1	56.0	54.3	55.8	56.0	54.1
N total (% DM)	8.1	8.7	8.5	9.4	9.7	8.9	9.2
C:N Ratio	6.9	6.5	6.6	5.8	5.8	6.3	5.9

Note: DM = dry matter. For C total, N total, and C:N ratio, only mean values are reported (n = 2). Different letters indicate significant differences (*p* < 0.05).

**Table 6:** Fatty acid profile (% of total fatty acids) of larvae reared on different feed substrates (mean ± SD).

	L1	L2	L3	L4	L5	L6	L7
C12:0	0.18 ± 0.02	0.17 ± 0.03	0.14 ± 0.04	0.15 ± 0.02	0.16 ± 0.02	0.19 ± 0.02	0.14 ± 0.02
C14:0	1.96 ± 0.21 ^ab^	1.75 ± 0.01 ^b^	1.65 ± 0.27 ^b^	1.69 ± 0.11 ^b^	1.90 ± 0.10 ^ab^	2.24 ± 0.04 ^a^	1.66 ± 0.03 ^b^
C15:0	0.08 ± 0.00 ^ab^	0.09 ± 0.01 ^ab^	0.08 ± 0.01 ^b^	0.10 ± 0.00 ^ab^	0.09 ± 0.01 ^ab^	0.10 ± 0.02 ^ab^	0.11 ± 0.01 ^a^
C16:0	18.78 ± 0.35	19.22 ± 0.64	18.30 ± 1.76	18.64 ± 1.31	18.48 ± 0.72	18.84 ± 1.04	17.72 ± 0.58
C16:1 (Δ9)	1.29 ± 0.09 ^b^	1.19 ± 0.11 ^b^	1.14 ± 0.14 ^b^	1.13 ± 0.21 ^b^	1.34 ± 0.05 ^b^	1.99 ± 0.08 ^a^	1.44 ± 0.08 ^b^
C16:2 (Δ7, Δ10)	0.16 ± 0.03 ^ab^	0.16 ± 0.01 ^b^	0.16 ± 0.03 ^ab^	0.19 ± 0.02 ^ab^	0.18 ± 0.03 ^ab^	0.14 ± 0.02 ^b^	0.22 ± 0.00 ^a^
C17:0	0.07 ± 0.01 ^b^	0.09 ± 0.02 ^ab^	0.07 ± 0.00 ^b^	0.10 ± 0.02 ^ab^	0.08 ± 0.02 ^b^	0.11 ± 0.01 ^ab^	0.13 ± 0.02 ^a^
C18:0	2.12 ± 0.35	2.62 ± 0.31	2.02 ± 0.47	2.67 ± 0.93	2.22 ± 0.45	2.29 ± 0.55	2.80 ± 0.63
C18:1 (Δ9)	43.11 ± 1.09 ^ab^	39.45 ± 0.93 ^bc^	38.70 ± 2.88 ^bc^	36.43 ± 4.57 ^c^	40.44 ± 1.18 ^abc^	46.12 ± 1.90 ^a^	39.87 ± 1.59 ^abc^
C18:1 (Δ11)	0.67 ± 0.12	0.65 ± 0.11	0.73 ± 0.08	0.74 ± 0.02	0.78 ± 0.14	0.79 ± 0.08	0.75 ± 0.09
C18:2 (Δ9, Δ12)	30.69 ± 0.28 ^ab^	33.40 ± 1.00 ^a^	35.80 ± 5.15 ^a^	36.84 ± 2.66 ^a^	33.15 ± 0.68 ^a^	26.43 ± 0.38 ^b^	34.41 ± 0.84 ^a^
C18:3 (Δ9, Δ12, Δ15)	0.89 ± 0.09 ^bc^	1.22 ± 0.06 ^ab^	1.21 ± 0.28 ^ab^	1.32 ± 0.14 ^a^	1.18 ± 0.04 ^ab^	0.75 ± 0.07 ^c^	0.75 ± 0.06 ^c^
SFA	23.11 ± 0.69	23.84 ± 0.97	22.19 ± 2.52	23.25 ± 2.14	22.83 ± 1.25	23.68 ± 1.60	22.44 ± 1.20
MUFA	45.23 ± 1.04 ^ab^	41.45 ± 1.10 ^b^	40.73 ± 2.93 ^b^	38.49 ± 4.79 ^b^	42.74 ± 1.25 ^ab^	49.04 ± 2.00 ^a^	42.28 ± 1.73 ^ab^
PUFA	31.66 ± 0.35 ^ab^	34.71 ± 1.04 ^a^	37.08 ± 5.43 ^a^	38.26 ± 2.80 ^a^	34.43 ± 0.72 ^a^	27.28 ± 0.44 ^b^	35.27 ± 0.88 ^a^
n-6/n-3	33.03 ± 4.86 ^b^	25.44 ± 0.66 ^c^	28.25 ± 2.14 ^bc^	26.13 ± 0.71 ^c^	26.04 ± 0.61 ^c^	31.28 ± 2.98 ^bc^	39.97 ± 1.44 ^a^

Note: SFA = saturated fatty acids; MUFA = monounsaturated fatty acids; PUFA = polyunsaturated fatty acids; n-6/n-3 = ratio of total n-6 to n-3 polyunsaturated fatty acids. Different letters indicate significant differences (*p* < 0.05).

**Table 7:** Macro and trace element concentrations of mealworm larvae reared on different feed substrates (mg/kg DM).

	L1	L2	L3	L4	L5	L6	L7
As	<LOD	<LOD	<LOD	<LOD	<LOD	<LOD	<LOD
Ca	292	396	478	382	407	401	437
Cd	<LOQ	<LOD	<LOD	<LOQ	<LOD	<LOQ	<LOQ
Cr	0.07	0.08	0.08	0.10	0.12	0.09	0.15
Cu	21.4	17.4	16.5	18.9	19.6	18.5	22.8
Fe	66.0	59.7	59.5	65.4	65.5	63.0	64.1
K	7414	7273	7569	8281	7999	7927	8666
Mg	2033	2060	2095	2436	2314	2358	2444
Mn	14.9	10.9	11.4	15.3	14.5	13.9	12.0
Ni	0.91	LOQ	0.59	0.60	1.23	1.02	1.20
P	7680	7754	7965	8764	8649	8772	9378
Pb	<LOQ	<LOD	<LOD	<LOD	<LOD	<LOD	<LOD
S	3236	3186	3229	3536	3586	3449	3628
Zn	118.1	111.3	109.3	135.2	126.9	127.1	132.5

Note: Values < LOD or <LOQ correspond to the limits listed in Table 3.

**Table 8:** Composition of mealworm frass from larvae reared on different feed substrates (mean ± SD).

	F1	F2	F3	F4	F5	F6	F7
Moisture %	18.4 ± 1.3 ^ab^	16.9 ± 1.4 ^ab^	15.9 ± 0.7 ^b^	16.0 ± 0.6 ^ab^	19.3 ± 1.6 ^a^	18.8 ± 1.3 ^ab^	15.8 ± 0.8 ^b^
Ash (% DM)	7.6 ± 0.1 ^a^	6.2 ± 0.5 ^bc^	6.7 ± 0.1 ^b^	5.1 ± 0.1 ^d^	5.4 ± 0.2 ^cd^	5.5 ± 0.6 ^cd^	6.9 ± 0.2 ^ab^
C total (% DM)	35.6	36.9	36.2	38.0	38.5	37.1	38.6
N total (% DM)	2.0	2.8	2.7	2.7	3.2	3.1	3.1
C:N Ratio	17.7	13.2	13.5	14.3	12.1	11.9	12.4

Note: DM = dry matter. For C total, N total, and C:N ratio, only mean values are reported (n = 2). Different letters indicate significant differences (*p* < 0.05).

**Table 9:** Macro and trace elements concentrations of mealworm frass from larvae reared on different feed substrates (mg/kg DM).

	F1	F2	F3	F4	F5	F6	F7
As	<LOD	<LOD	<LOD	<LOD	<LOD	<LOD	<LOD
Ca	1345	2184	2179	1870	1898	983	2950
Cd	<LOQ	<LOQ	<LOQ	<LOQ	<LOQ	<LOQ	<LOQ
Cr	0.18	0.26	0.25	0.26	0.26	0.24	1.32
Cu	13.4	13.5	12.1	12.0	11.9	8.6	23.0
Fe	211.4	228.4	206.6	192.2	193.5	139.3	507.2
K	12,990	10,137	10,130	9084	9805	11,896	14,324
Mg	4993	4251	4033	3770	3534	2822	3061
Mn	209.2	165.2	149.8	138.9	139.0	98.7	114.9
Ni	2.8	1.9	1.7	1.4	1.5	1.6	3.4
P	11,501	9679	9241	8983	9217	9366	9364
Pb	<LOD	<LOQ	<LOD	<LOD	<LOD	0.59	0.55
S	2118	2551	2313	2356	2583	2078	2366
Zn	97.6	88.4	79.9	80.8	78.7	45.4	52.5

Note: Values < LOD or <LOQ correspond to the limits listed in Table 3.

## Data Availability

The original contributions presented in the study are included in the article/Supplementary Material, further inquiries can be directed to the corresponding authors.

## Supplementary Materials

The following supporting information can be downloaded at: https://www.mdpi.com/article/10.3390/foods15091478/s1. Table S1: Fatty acid profile (% of total fatty acids) of substrate treatments (mean ± SD); Table S2: Amino acid profile (% of total identified amino acids) of substrate treatments.
