# Supplementary material for: Upcycling Agri-Food Side-Streams via Tenebrio molitor Rearing: Growth Performance, Nutritional Composition, and Frass Quality of Larvae
Source: Foods. 2026 Apr 23;15(9):1478. doi: 10.3390/foods15091478 (PMC13164134; doi:10.3390/foods15091478)
Supplement: Supplementary file 1 [file foods-15-01478-s001.zip › foods-4232846-supplementary.pdf]

Table S1: Fatty acid profile (% of total fatty acids) of substrate treatments (mean ± SD).

	**S1**	**S2–4**	**S5**	**S6**	**S7**
C8:0	ND	0.13 ± 0.06	0.10 ± 0.07	0.10 ± 0.01	0.07 ± 0.01
C10:0	ND	1.52 ± 0.18	1.48 ± 0.13	0.87 ± 0.09	0.65 ± 0.06
C12:0	ND	0.21 ± 0.07	0.21 ± 0.05	0.12 ± 0.04	0.10 ± 0.03
C14:0	ND	ND	0.02 ± 0.01	0.07 ± 0.03	0.08 ± 0.02
C16:0	19.75 ± 0.27	27.62 ± 0.16	27.54 ± 0.24	25.39 ± 0.48	21.12 ± 0.28
C16:1 (Δ9)	ND	8.67 ± 0.10	8.48 ± 0.31	5.08 ± 1.05	3.91 ± 0.77
C16:1 (Δ11)	ND	ND	0.02 ± 0.00	0.07 ± 0.02	0.05 ± 0.01
C17:0	ND	ND	ND	0.10 ± 0.01	ND
C18:0	0.56 ± 0.01	2.81 ± 0.33	3.03 ± 0.43	4.14 ± 0.84	3.21 ± 0.65
C18:1 (Δ9)	17.08 ± 0.25	14.22 ± 0.50	14.25 ± 0.50	14.69 ± 0.71	16.63 ± 0.49
C18:1 (Δ11)	1.20 ± 0.06	0.84 ± 0.04	0.85 ± 0.05	0.88 ± 0.18	1.04 ± 0.11
C18:2 (Δ9, Δ12)	58.52 ± 0.41	42.00 ± 0.31	42.00 ± 0.27	44.67 ± 1.85	51.25 ± 0.48
C18:3 (Δ9, Δ12, Δ15)	2.89 ± 0.19	2.02 ± 0.13	2.02 ± 0.13	3.46 ± 0.21	1.89 ± 0.14
C20:0	ND	ND	ND	0.60 ± 0.12	ND
SFA	20.31 ± 0.29	32.25 ± 0.00	32.37 ± 0.15	31.16 ± 1.12	25.23 ± 0.75
MUFA	18.28 ± 0.31	23.73 ± 0.44	23.60 ± 0.36	20.72 ± 1.11	21.63 ± 0.32
PUFA	61.41 ± 0.60	44.02 ± 0.45	44.03 ± 0.40	48.13 ± 2.01	53.14 ± 0.55
n-6/n-3	20.30 ± 1.19	20.82 ± 1.21	20.82 ± 1.23	12.93 ± 0.52	27.19 ± 1.87

Note: ND = not detected. SFA = saturated fatty acids; MUFA = monounsaturated fatty acids; PUFA = polyunsaturated fatty acids; n-6/n-3 = ratio of total n-6 to n-3 polyunsaturated fatty acids. Different letters indicate significant differences ($p < 0.05$).

Table S2: Amino acid profile (% of total identified amino acids) of substrate treatments.

	**S1**	**S2–4**	**S5**	**S6**	**S7**
HIS	3.57	3.16	3.20	3.40	3.54
ILE	3.53	3.72	3.80	4.11	4.05
LEU	6.88	7.05	7.05	7.10	7.06
LYS	4.87	4.39	4.74	6.17	5.93
MET	1.68	2.04	2.01	1.84	1.80
PHE	4.53	5.06	4.97	4.61	4.62
THR	3.75	3.62	3.80	4.52	4.39
TRP	1.17	1.32	1.29	1.11	1.01
VAL	5.17	5.17	5.22	5.43	5.39
Total EAA	35.14	35.52	36.07	38.29	37.79
ALA	5.42	4.89	5.15	6.15	5.84
ARG	7.20	6.13	6.19	6.37	6.56
ASP	7.99	7.27	7.61	9.37	8.84
CYS	2.52	3.16	2.99	2.19	2.26
GLU	21.70	22.42	21.66	18.42	18.97
GLY	5.77	4.89	4.98	5.40	5.84
PRO	6.91	8.47	7.98	5.86	6.00
SER	4.99	4.70	4.81	5.29	5.20
TYR	2.35	2.54	2.58	2.67	2.69
Total NEAA	64.86	64.48	63.93	61.71	62.21
EAA/NEAA	0.54	0.55	0.56	0.62	0.61

Note: Only mean values are reported due to the limited number of replicates (n = 2).
